# Global ozone loss following extreme solar proton storms based on the July 2012 coronal mass ejection

**DOI:** 10.1038/s41598-023-40129-1

**Published:** 2023-08-24

**Authors:** Niilo Kalakoski, Pekka T. Verronen, Monika E. Szeląg, Charles H. Jackman

**Affiliations:** 1 Space and Earth Observation Centre, Finnish Meteorological Institute, Helsinki, Finland; 2 Sodankylä Geophysical Observatory, University of Oulu, Sodankylä, Finland; 3 NASA Goddard Space Flight Center, Greenbelt, MD USA

**Keywords:** Natural hazards, Solar physics, Atmospheric chemistry, Climate and Earth system modelling

## Abstract

Large solar coronal mass ejections pose a threat in the near-Earth space. As a cause of extreme periods of space weather, they can damage satellite-based communications and create geomagnetically induced currents in power and energy grids. Further, the solar wind energetic particles can reduce the protecting layer of atmospheric ozone and pose a threat to life on Earth. The large coronal mass ejection (CME) of July 2012, although directed away from the Earth, is often highlighted as a prime example of a potentially devastating super storm. Here we show, based on proton fluxes recorded by the instruments aboard the STEREO-A satellite, that the atmospheric response to the July 2012 event would have been comparable to those of the largest solar proton events of the satellite era. Significant impact on total ozone outside polar regions would require a much larger event, similar to those recorded in historical proxy data sets. Such an extreme event would cause long-term ozone reduction all the way to the equator and increase the size, duration, and depth of the Antarctic ozone hole. The impact would be comparable to predicted drastic and sudden ozone reduction from major volcanic eruptions, regional nuclear conflicts, or long-term stratospheric geoengineering.

## Introduction

Solar proton events (SPEs) are caused by the flux of charged particles, mainly protons, accelerated in the solar corona by the Sun’s magnetic field during coronal mass ejections. The particles generally have energies in the range of tens to hundreds of megaelectronvolts (MeV). Guided by the Earth’s magnetic field, protons can penetrate deep into the atmosphere, influencing the composition of atmosphere via ionization and dissociation of neutral species. Increased production of odd hydrogen and nitrogen species is shown to influence the ozone depletion in polar mesosphere and upper stratosphere^1–4^. This short-term ozone variability can also affect the temperature of the middle atmosphere^5^, possibly modulating the polar winter dynamics on decadal timescales^6–8^.

Variability of upper stratospheric ozone due to SPE-induced losses is also one of the factors affecting the long-term ozone recovery expected after the reduction of emission of anthropogenic ozone depleting substances^9,10^. The impact of the solar proton event on the stratospheric ozone and subsequently on the total column ozone is expected to result from the so-called indirect effect driven by ${\hbox {NO}_y}$ transported from the mesosphere, rather than the direct effect driven by the highest particle energies^11^. During the satellite era, largest SPEs have reduced total ozone column in the polar regions at most by a few percent for a period of a few months^12–14^.

In the modern era the most famous solar storm, so-called Carrington event, occurred in September 1859. While detailed observations for this event are not available, it was one of the largest space weather event in recorded history. Modelling studies of the atmospheric effects indicate a qualitatively similar, but stronger response than those observed for the strongest events of the satellite era. A consistent feature in these studies is a long-lasting ozone depletion in the middle and upper stratosphere, with a maximum depletion about 2 months after the event^15^.

Evidence of even more extreme SPEs is available from the long-term records of cosmogenic isotopes (mainly ${^{14}\hbox {C}}$ and ${^{10}\hbox {Be}}$). The anomaly seen in these records for years 774–775 CE is likely due to an extremely large SPE, possibly 25–50 times stronger than the SPE event of 23 Feb. 1956^16,17^. Events of a similar magnitude are suggested for years around 660 BCE^18^ and for 993 CE^19^. In the historical proxy records, the 774–775 CE event likely represents the worst case scenario for the holocene^20^. As the prevailing atmospheric conditions can greatly affect the impact of the SPEs, direct comparisons to historical events are not straightforward^21^. The middle atmospheric chemistry effects can be intensified and observed for months or even years.

Solar storms of the satellite era, where the observations of space weather and the state of the atmosphere are readily available, have been extensively studied. The solar cycles 22 and 23 (1986–2008) featured several strong storms that caused damage to satellites and power grids, raising concerns over the vulnerability of the infrastructure to extreme solar storms.

During an otherwise quiet Solar cycle 24 (2008–2019), large coronal mass ejection occurred at 23 July 2012. While directed away from the Earth, the CME was observed by the STEREO-A satellite^22^ orbiting the Sun at roughly 1 AU distance, preceding the Earth by 121.3$^{\circ }$^23^. Due to the preconditioning of the solar wind by an earlier (19 July) solar eruption, the transit time of the disturbance to STEREO-A was unusually fast. Together with high particle fluxes the disturbance was suggested to have produced a strong geomagnetic period, comparable to the strongest events of satellite era had it hit the Earth^23–25^. Based on the space weather forecast methods, Baker et al.^26^ estimated that the potential geomagnetic storm produced by July 2012 CME would have been comparable to largest storms of the 20th century (about − 500 nT measured with the geomagnetic Dst index, compared to e.g. − 583 nT for March 1989 and − 383 nT for Halloween storm of 2003^27^). By varying the season and the orientation of the magnetic field the most extreme plausible model scenario produced a geomagnetic disturbance with Dst = − 1182 nT, within the range of the estimates proposed for the Carrington storm of 1859. (e.g.^28,29^) The potential impact of the July 2012 event on terrestrial electrical systems and satellite communications were widely speculated on at the time. However, the potential effects of the event on the atmospheric constituents have not been studied in depth.

Some smaller, but notable solar proton events hit the Earth in early 2012 (23–30 January and 7–11 March 2012). These included the highest proton fluxes recorded during the otherwise quiet Solar cycle 24^14,30^. The 2012 Antarctic ozone hole was relatively small and a strong rebound was reported in October and November 2012 (e.g.^31^) with a split of the ozone hole occurring in November. In the Northern Hemisphere, the Winter 2012/13 was characterized by a sudden stratospheric warming starting in December 2012 (e.g.^32^).

In this paper, we simulate the ozone response to the proton fluxes observed by the STEREO-A instrument in July 2012. After analysing the STEREO-A observations, we find the proton fluxes for July 2012 to be equal to the largest SPEs of the satellite era. Further, by increasing the July 2012 fluxes by up to a factor of hundred, to match the extreme fluxes derived from the historical proxy records, we are the able to assess the feasible, historical range of total ozone responses from extremely large events. Finally, we increase the STEREO fluxes by a factor of thousand to test for a possible saturation of the ozone impact. Because we are assessing the impact from a range of extreme events, we mainly look at changes in the total ozone column and how the impact is transmitted from polar regions to lower latitudes.

The model experiments include five simulations using Whole Atmosphere Community Climate Model (WACCM)^33,34^, with variable proton-induced ionization: REF simulation with no proton ionization throughout the simulation period and four simulations with CMIP6 proton forcing^35^ and an additional forcing for the event period (July 17–31, 2012), based on STEREO observations. The additional forcing was applied directly in one simulation and multiplied by a factor of 10, 100 and 1000 in three additional simulations. For further details on the simulations, see the “Methods” section).

## Results

**Figure 1:**
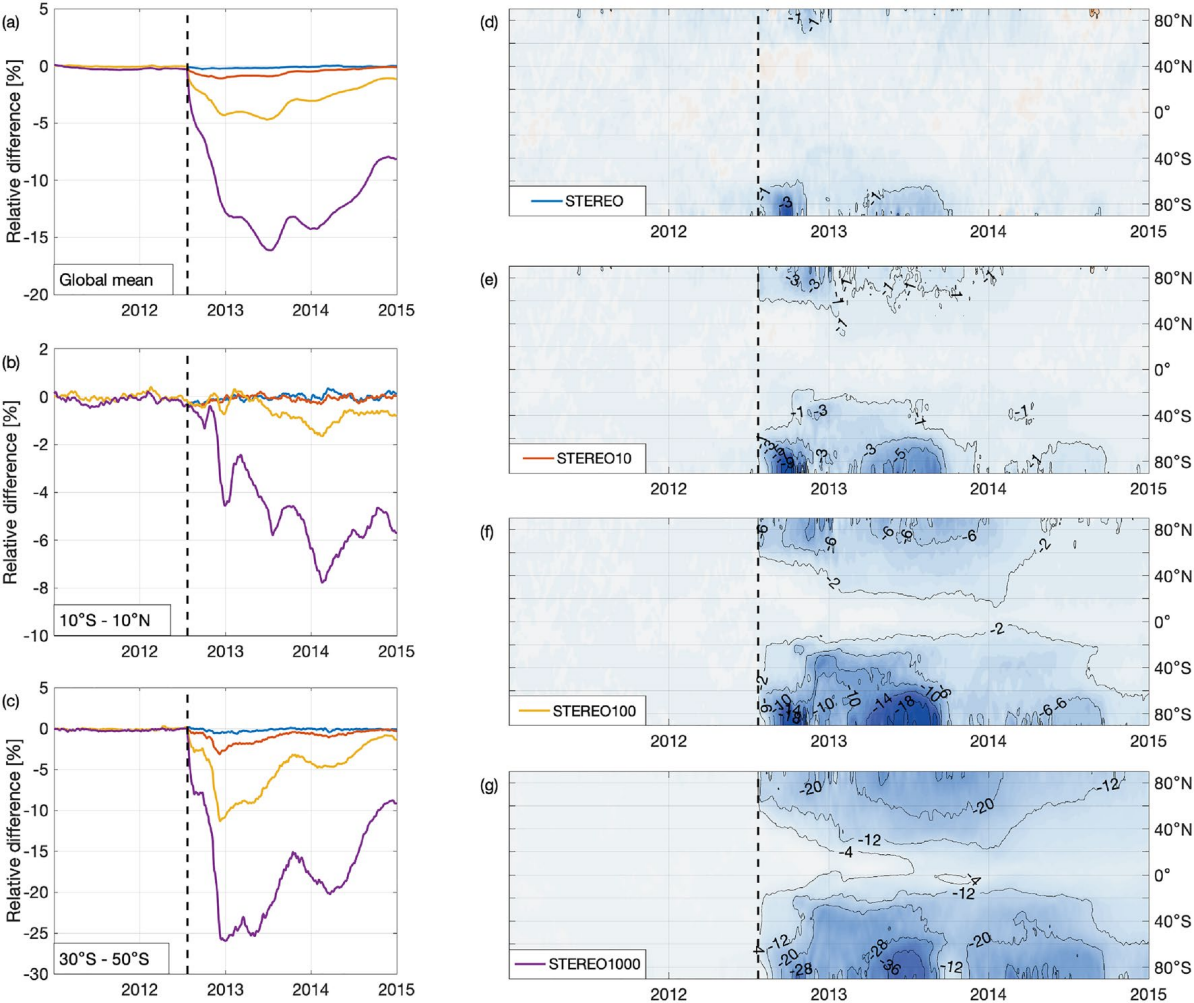
Time series of 7-day running mean zonal mean total ozone anomalies in percent. (**d**–**g**) Show the anomaly of the zonal mean total ozone for each latitude band in STEREO (**d**), STEREO10 (**e**), STEREO100 (**f**) and STEREO1000 (**g**) simulations. (**a**–**c**) show the global (**a**), equatorial (**b**) and southern mid-latitudes (**c**) mean ozone anomaly for each simulation. Note the different contour lines and color scales in panels (**d**–**g**).

As expected, strong solar proton events have a noticeable impact on the total ozone column (Fig. 1). In the simulation using STEREO proton forcing (panel d), zonal mean ozone anomalies in the southern polar latitudes show a decrease of more than 3% in September–October 2012, during the Southern Hemisphere ozone hole season. The seasonality and hemispherical asymmetry of the total ozone response is also clearly seen, with a weaker response occurring after a longer delay in the Northern Hemisphere. When the proton forcing measured by STEREO-A is increased by a factor of 10, 100 or 1000 (panels e–g), the total ozone response is enhanced, reaching maximum decreases of about 10%, 20% and 40%, respectively. In the Southern Hemisphere, a major ozone anomaly, regardless of the STEREO scenario, is initially largely confined within the polar cap. However, in STEREO10 and STEREO100 scenarios, the anomaly spreads rapidly to mid-latitudes following the breakup of the polar vortex in late 2012. A similar expansion of the affected area is also observed in the Northern Hemisphere in STEREO100 scenario starting in the beginning of 2013. Expansion in the Northern Hemisphere is likely related to the breakup of the northern polar vortex following the January 2013 SSW. In addition to the deep minimum observed in the polar areas, a persistent reduction of about 2$\%$ is observed in the STEREO100 simulation, quickly reaching tropical latitudes in the Southern Hemisphere and lasting until mid- to late 2014. STEREO1000 scenario shows features similar to those seen in the less extreme scenarios. The magnitude and the extent of the anomalies is however clearly larger. The deepest ozone depletion in the Southern hemisphere are about twice as strong as in STEREO100 scenario. The ozone loss in STEREO1000 is also global in extent with zonal means clearly below the reference at the end of simulation period.

As seen in the left panels of the Fig. 1, the ozone anomalies in the STEREO10 and STEREO100 scenarios are sufficient to be observed in the global mean total ozone columns with roughly 1$\%$ and 4–5$\%$ reductions in STEREO10 and STEREO100, respectively. While the greatest reductions are limited to polar areas, ozone reductions of several percents are also observed in mid-latitudes, persisting until at least year 2014. After a maximum reduction of about 15%, the STEREO1000 scenario is still 8% below the reference at the end of year 2014. In general, the ozone anomalies observed in STEREO1000 simulation are larger than those observed in STEREO100 by a factor of 2–3. This indicates that the chemical response is not saturated at the level of STEREO100 scenario, but an even more extreme ozone response would occur if an event at the STEREO1000 magnitude was possible.

**Figure 2:**
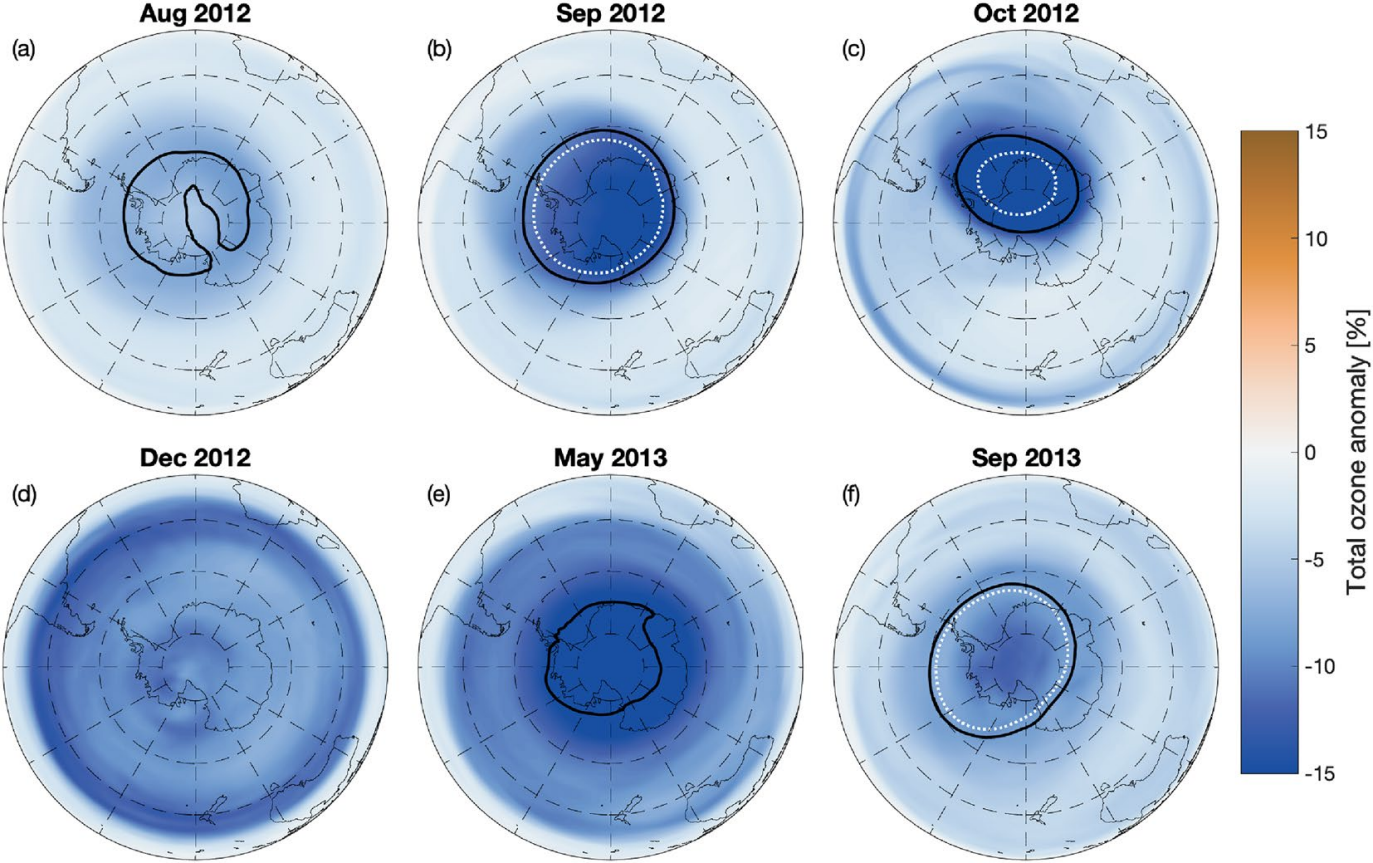
STEREO100 monthly mean total ozone anomaly maps (in percent) for selected months. Black solid lines in (**a**–**c**) and (**e**–**f**) represents the edge of the ozone hole (220 DU) in the STEREO100 simulation, while the white dotted lines in (**b**), (**c**) and (**f**) represents the edge of the ozone hole in the Reference simulation.

In the case of STEREO100, the most extreme historical scenario simulated, negative monthly mean ozone anomalies are seen throughout the Southern Hemisphere starting in August 2012 (Fig. 2). The ozone hole threshold of 220 DU is reached already in August, with the September–October ozone hole up to 20% (40 DU) deeper and somewhat larger in area than the hole observed in the reference simulation. As seen in the Fig. 1, the ozone anomalies expand to lower latitudes towards the end of the year. In December 2012, 10–15% relative ozone losses are seen around 40° S latitudes. The continued presence of elevated amounts of active nitrogen species then leads to low total ozone column amounts around the South Pole in early winter, reaching below 220 DU in April–May, several months before the normal season for ozone hole conditions. With the formation of the 2013 ozone hole around September, the ozone hole extent in the STEREO100 scenario is similar to the reference. However, low ozone anomalies persist throughout the Southern Hemisphere. As seen in the Fig. 1, an early winter anomaly, although weaker, recurs in the Southern Hemisphere in 2014. The ozone layer in the Southern Hemisphere finally shows signs of returning to a normal seasonal variation at the end of 2014. However, a negative anomaly of 1–2% persists in southern polar areas until the end of simulation period.

**Figure 3:**
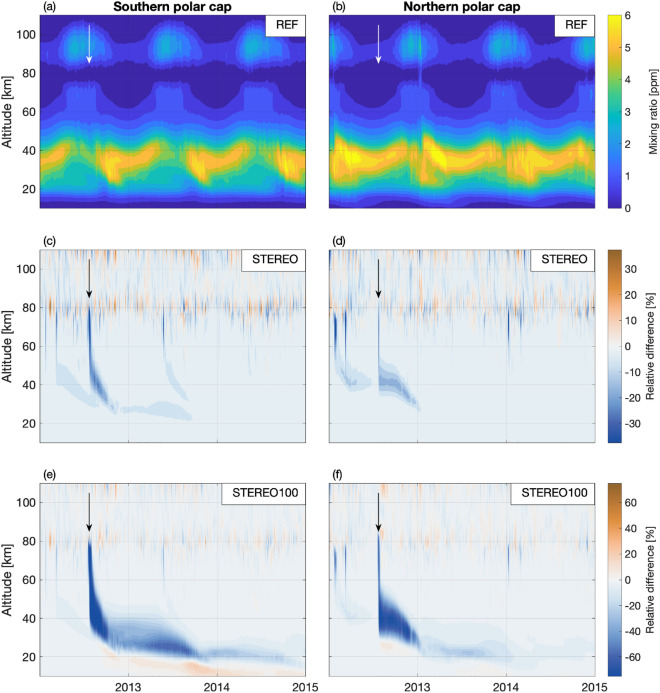
Polar cap (60–90$^{\circ }$) mean ozone profiles and anomalies. Daily mean time series (2012–2014) of polar cap average ozone mixing ratios from the reference simulation (**a**,**b**). Relative difference of polar cap averages (S-REF/REF) in percent for STEREO (**c**,**d**) and STEREO100 (**e**,**f**) simulations. Left panels (**a**,**c**,**e**) show the southern polar cap, while right panels (**b**,**d**,**f**) show the northern polar cap. Arrows mark the onset of the July 2012 event.

The vertical profiles of the ozone anomaly illustrate the importance of the season in which the SPE occurs (see Fig. 3). In scenarios shown (STEREO, STEREO100 and STEREO1000) the direct SPE effect is clearly seen immediately following the onset of July 2012 SPE ionization at pressure levels between 10 and 10$^{-2}$ hPa. The anomaly then gradually extends to lower levels during the next few months. The descent is noticeably faster in the Southern Hemisphere, where stronger downward transport associated with the winter conditions is present immediately following the July SPE. In the Northern Hemisphere, the summer conditions slow the downward propagation of the anomaly until the last months of 2012. Downward propagation is then further disrupted at the beginning of the year 2013 by the SSW^32^.

In the STEREO scenario the anomalies largely disappear by the end of 2012. Also apparent in the STEREO scenario is the hemispherical and seasonal asymmetry in the response. The response is clearly stronger in the Southern Hemisphere winter, than in the northern, summer pole. Likewise, in the Northern Hemisphere, the much weaker (in proton flux) historical events occurring in early 2012 produce a clearer direct response than the stronger July 2012 SPE. Ozone anomalies due to weak events in May 2013 and January 2014 are also visible in the Southern and Northern Hemispheres, respectively. In the lower stratosphere, depletion of active chlorine species by the descending ${\hbox {NO}_y}$ results in a net positive ozone anomaly^36^.

In the STEREO100 scenario, the anomalies are not only stronger but also persist for much longer than in the STEREO scenario. In the Southern Hemisphere, especially, the relative anomalies strengthen again after weakening during the summer ozone maximum, while continuing to descend towards the lower stratosphere (see Fig. 3). In STEREO100, anomalies recur in 2014. At this point, the anomaly is largely confined to the lower stratosphere. In the Northern Hemisphere, the timing of the descent and recurrence is different with the initial descent being completed in early 2013 and a much weaker recurrence occurring in mid-to-late 2013, before the northern winter.

Ozone anomalies are dominated by the odd-nitrogen species (${\hbox {NO}_y}$), created by the ionization in mesosphere and upper stratosphere. These relatively long-lived species are then transported to lower levels. Active chlorine species, which in normal circumstances play an important role in the reactions destroying ozone during polar winter, are also themselves destroyed by the increased ${\hbox {NO}_y}$ amounts in the aftermath of a massive SPE. While this reduction of active chlorine species helps to reduce the amount of ozone lost during the winter^36^, the increase of ${\hbox {NO}_y}$ is large enough to produce the losses seen in the stratosphere.

## Discussion

A scenario based on the STEREO proton fluxes produces a chemical response similar to previously studied historical SPEs (e.g.^4^). Ozone loss on a global scale seems to require an extraordinarily powerful SPE, similar in scale to STEREO10 or STEREO100 scenarios presented here. Even in the most extreme historical scenario, the effect is transitory (2–3 years), with predicted maximum global mean total ozone loss of about 4% (see Fig. 1a). However, the impact on total ozone varies regionally, depending on the timing of the event. The maximum ozone loss observed at southern mid-latitudes (around 10$\%$) occurs in December, coinciding with the already high UV radiation in inhabited areas such as South America or Southern Australia, highlighting potential health hazards of the extreme ozone losses. Based on the Radiative Amplification Factors based on the surface observations presented by McKenzie et al.^37^, at mid-latitudes 10% decrease of total ozone corresponds to a UV index increase of about 13%. The increase is however sensitive to the action spectrum weighting selected for UV. The DNA damage weighted radiation, for example, increases by around 25% for the same 10% reduction of total ozone. Therefore, following the ozone losses shown in the STEREO100 scenario, the anomalous UV radiation doses would be expected to result in severe health hazards (e.g. increase of incidence of skin cancer and cataracts), as well as damage to ecosystems (e.g. suppression of photosynthetic rates)^38^.

STEREO100 scenario uses a proton flux that is of a comparable scale to the 774–775 CE SPE event, which can be considered a realistic upper limit for the solar eruptions in our current knowledge. Usoskin and Kovaltsov^39^ give an estimated occurrence rate of 1–2 times per millennia or rarer for solar eruptions of this magnitude. Thomas et al.^40^ discuss the terrestrial effects of the 774–775 CE event with different projected energy spectra, concluding that for the most realistic scenarios, the effects on life would be small to moderate.

Notably the hemispherical asymmetry of the response in July 2012 event is opposite to the response modeled for the Carrington event 1859^41^ or the 774–775 CE event. In these studies, the SPE occurred in the beginning of September, with largest ozone losses in following months occurring in the Northern Hemisphere. The difference illustrates the importance of timing (i.e. amount of sunlight and different dynamical conditions) of the event to the geographical distribution of the response. For the SPE occurring in mid-July, the relative lack of sunlight and the strong downward transport in the Southern Hemisphere allows the descent of ${\hbox {NO}_y}$ produced by the SPE and the subsequent destruction of upper stratospheric ozone. For the events initiated in early September or later in the year, the conditions are starting to reverse, favouring the accumulation and transport of ${\hbox {NO}_y}$ in the Northern Hemisphere. It should also be kept in mind that unlike these studies of historical events^17,41^, our simulations consider a case with a ozone layer weakened as a result of the anthropogenic emissions of ozone-depleting substances.

As a case study, we choose to describe the events in the framework of the historical 2012 dynamical conditions. As such, the dynamical changes incurred by the chemical changes are not taken into account. However, the largest anomalies are observed in the Southern Hemisphere winter, where the strong polar vortex dominates the dynamical conditions. While large, the ozone anomalies are likely not strong enough to severely disrupt the vortex beyond relatively minor changes in the timing of the breakup of the vortex. Dynamical context of the response is however strongly dependent on the timing of the studied event. In the case of STEREO1000 scenario, it seems likely that due to the extreme and long lasting ozone anomalies seen, the inclusion of interactive dynamics would be necessary for the accurate description of such a “post-realistic” scenario. The changes in the radiative balance and subsequently temperature gradients within southern hemisphere polar vortex could be expected to lead to strengthening of the polar vortex, resulting in its delayed breakup and anomalous troposheric circulation throughout the hemisphere (e.g.^42^). The dynamical aspects of these colossal events present an interesting opening for a follow-up study.

In addition to the millennial scale solar storms, several other potentially catastrophic sources of large sudden ozone losses have been put forward in earlier studies. Large volcanic eruptions, occurring in tropical latitudes, have been shown to be capable of reducing ozone total columns by up to 20% globally, producing ozone hole conditions (total columns below 220 DU) in the tropics. Depending on the assumptions on eruption parameters, the ozone layer can take up to 10 years to recover^43^ following a large eruption. Similar ozone losses can result from the stratospheric injection of soot from fires following a regional scale nuclear conflict^44^. In contrast, radiation belt remediation after a single high altitude nuclear explosion is expected to induce ozone losses of a similar magnitude to relatively large SPEs, persisting for a few days^45^. Sulfur injection strategies proposed to counter greenhouse gas induced warming can potentially deepen the Antarctic ozone hole by 8–20%, delaying the ozone recovery by 25–55 years^46^. In longer timescales, the weakening of the Earth’s magnetic field and the following magnetic pole reversal has been proposed to lead to global reduction and redistribution of stratospheric ozone concentration and subsequent changes in atmospheric circulation^47,48^. As seen in the STEREO100 scenario, the extreme solar storms are capable of producing ozone losses of similar scale to other potential events described above. Another common theme for the different sources is the uncertainty of the timescales involved and the difficulty of predicting future events.

As a conclusion, from the middle atmosphere chemistry point of view, the proton fluxes produced by July 2012 coronal mass ejection would not have been unprecedented compared to the largest recorded SPEs of the satellite era. even though the storm had the potential to cause an unprecedented geomagnetic storm, the ionization rates, and consequently chemical impact would not have been larger than the ones observed during Solar cycles 22 and 23. Based on the results of the extrapolated STEREO10 and STEREO100 simulations, significant total ozone losses with global distribution would result from an extreme but realistic event. While the events of this magnitude are rare, the conditions in which they occur are not well known and the prediction of the occurrence is currently not possible. As such, the main dangers of the catastrophic solar storms likely rest elsewhere, in the damage caused to the man-made systems such as terrestrial energy infrastructure or satellite communications. Nevertheless, the response predicted by the STEREO100 scenario points to significant global ozone loss lasting a few years.

## Methods

### Data sources and modelling

Whole Atmosphere Community Climate Model Version 6 (WACCM6) is a atmospheric part of Community Earth System Model Version 2 (CESM2). It is state of the art chemistry-climate model with horizontal resolution 0.9$^{\circ }$ latitude by 1.25$^{\circ }$ longitude, 88 pressure levels and the model top at about 6 $\times$ 10$^{-6}$ hPa ($\sim$140 km). WACCM6 incorporates a comprehensive chemistry mechanism that is relevant to investigate interactions across the whole atmosphere. The features of the model and comparison with observations are described in details in Gettelman et al.^33^. Here, we use WACCM6 in the specified dynamics configuration (FWmadSD), which nudges horizontal winds and temperatures below 50 km altitude towards the Modern-Era Retrospective analysis for Research and Applications (MERRA2^49^). This configuration reduces climate noise, wind and temperature biases and is well suited for reproducing the chemical response to the specific events such as SPEs. We utilize the middle atmosphere D-region (MAD) chemical mechanism, which includes negative ion and cluster ion chemistry^34^.

For the model runs we used the recommended CMIP6 (Coupled Model Intercomparison Project Phase 6) solar and geomagnetic forcing^35^, except that we apply four different scenarios for ion-pair production rates by solar protons: a reference run with zero proton ionization, and three simulations with CMIP6 proton forcing and different levels of added forcing on July 17–31, 2012, based on STEREO observations (see Table 1).

**Table 1:** Solar proton forcing applied in four different model experiments.

Set	Description	Proton ionization
REF	Reference run	0
STEREO	Scenario 0	CMIP6 + STEREO
STEREO10	Scenario 1	CMIP6 + 10 x STEREO
STEREO100	Scenario 2	CMIP6 + 100 x STEREO
STEREO1000	Scenario 3	CMIP6 + 1000 x STEREO

All other solar forcing, both particles and irradiance, is from the CMIP6 recommendation.

**Figure 4:**
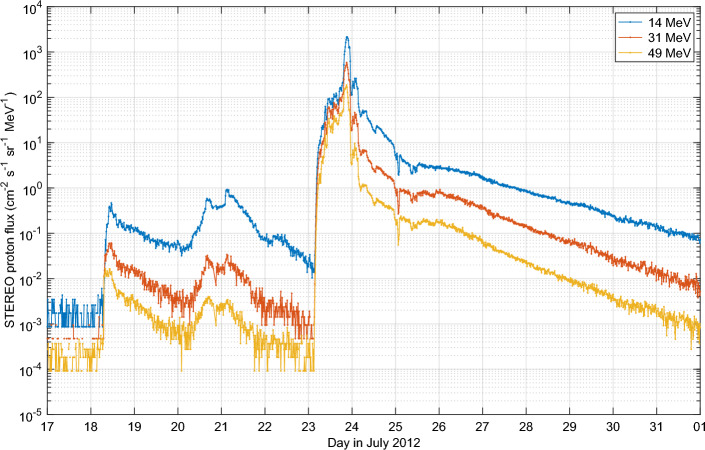
STEREO proton fluxes, measured in July 2012. Three proton energy are shown.

The STEREO-A instruments provide us proton fluxes at keV-to-MeV energy range for the July 2012 event (https://izw1.caltech.edu/STEREO, accessed in 15 February 2023). As an example, Fig. 4 shows the measured fluxes for three high proton energies. A daily average proton energy-flux spectrum was combined from STEREO-A measurements, for a continuous spectrum in proton energies between 1 and 300 MeV which correspond to direct proton impact at altitudes between about 90 and 25 km, respectively. Note that STEREO measures energies up to $\sim$80 MeV, so the fluxes at energies higher than that are extrapolated.

The ionization rate calculation follows a continuously slowing-down approximation approach presented in detail by Verronen et al.^50^, was originally presented by Reid^51^, and is based on an empirical energy-range relation for protons^52^.

**Figure 5:**
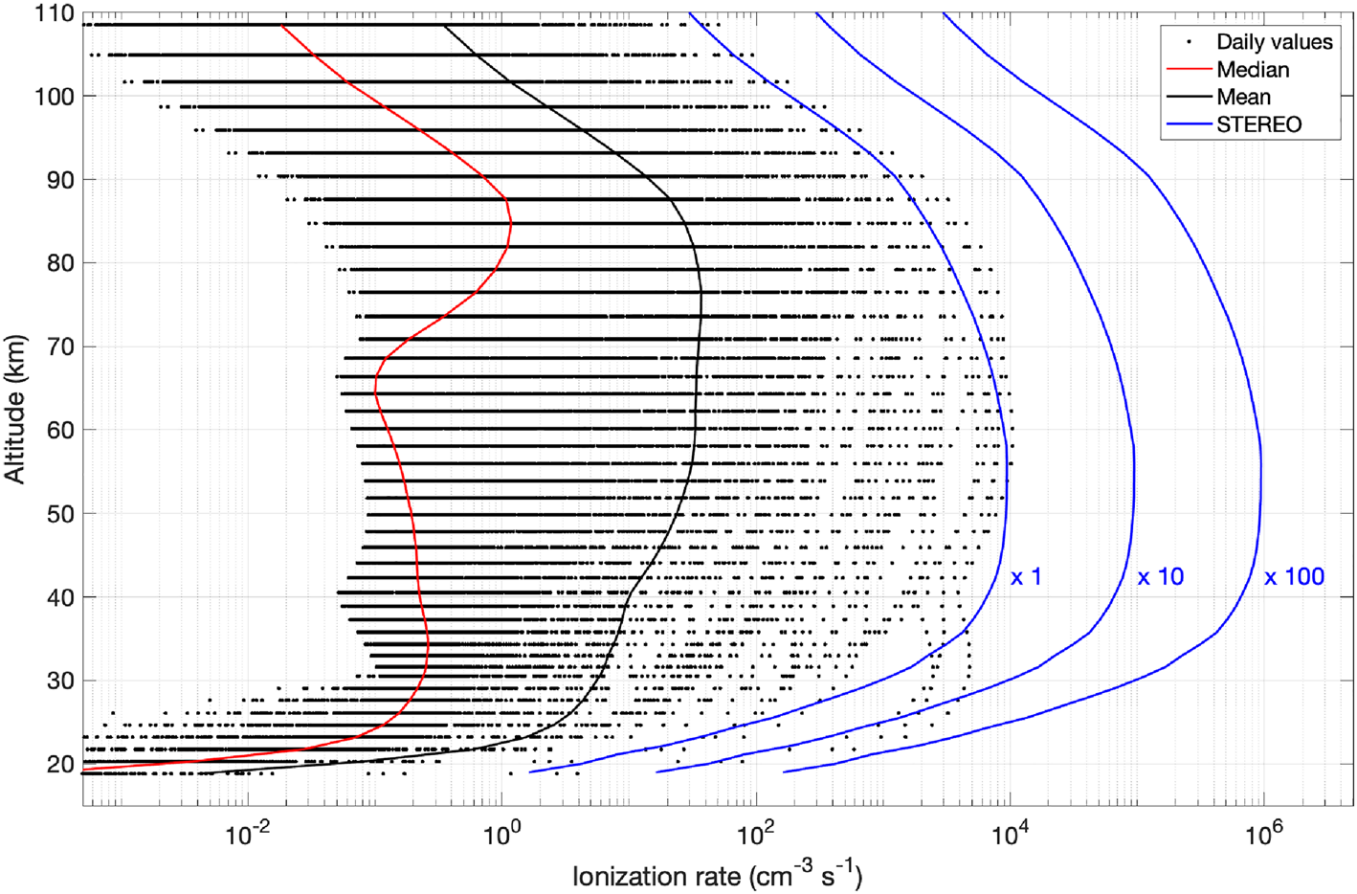
Comparison of ionization rate profiles of STEREO scenarios with daily, mean and median CMIP6 proton ionization profiles in 2000–2005.

The peak ionization rates from 23rd July are compared to daily CMIP6 proton ionization rates from years 2000–2003 in Fig. 5. Clearly, the STEREO rates are comparable to those of the strongest solar proton events, such as July 2000 and October 2003, but only exceed them at a limited altitude range around 40 km. Below 30 km, which corresponds to the extrapolated high-energy tail of the spectrum, STEREO rates are only a fraction of those of largest events. Figure 5 also shows that by multiplying the STEREO rates by 10 and 100, we exceed the largest recorded events in 2000–2003 by approximately the same ratio in the upper stratosphere and above. The comparisons in the Fig. 5 should not be considered exact, due to the different instrumentation (i.e. different energy channels) and different observational environments (i.e. geostationary orbit vs. solar orbit). While formal inter-calibration of particle detectors aboard GOES and STEREO satellites are not available, the available comparisons^53^ indicate that the integral fluxes (as used here) from these instruments agree well. Likewise, the shielding effect of Earth’s magnetic field at the geomagnetic orbit is relatively weak for the high energy protons, which are the most relevant for the middle atmosphere ionization (see e.g.^54,55^).

## Data Availability

The simulation data analysed in this paper will be available at Finnish Meteorological Institute Research Data repository METIS (http://hdl.handle.net/11304/2a712120-4f77-462e-b1b6-d4e7f13e03e5).
